# P-169. Acute Organ Failure and Death among Hospitalized Adults with Babesiosis

**DOI:** 10.1093/ofid/ofaf695.393

**Published:** 2026-01-11

**Authors:** Audrey E Monson, Peter J Krause, David E Leaf

**Affiliations:** Brigham and Women's Hospital, Lewisburg, WV; Yale School of Public Health and Yale School of Medicine, Sudbury, Massachusetts; Brigham and Women's Hospital, Lewisburg, WV

## Abstract

**Background:**

Babesiosis is an emerging tickborne illness that may result in acute organ injury/failure and death. However, detailed data on severe illness from babesiosis are limited.

**Figure:**
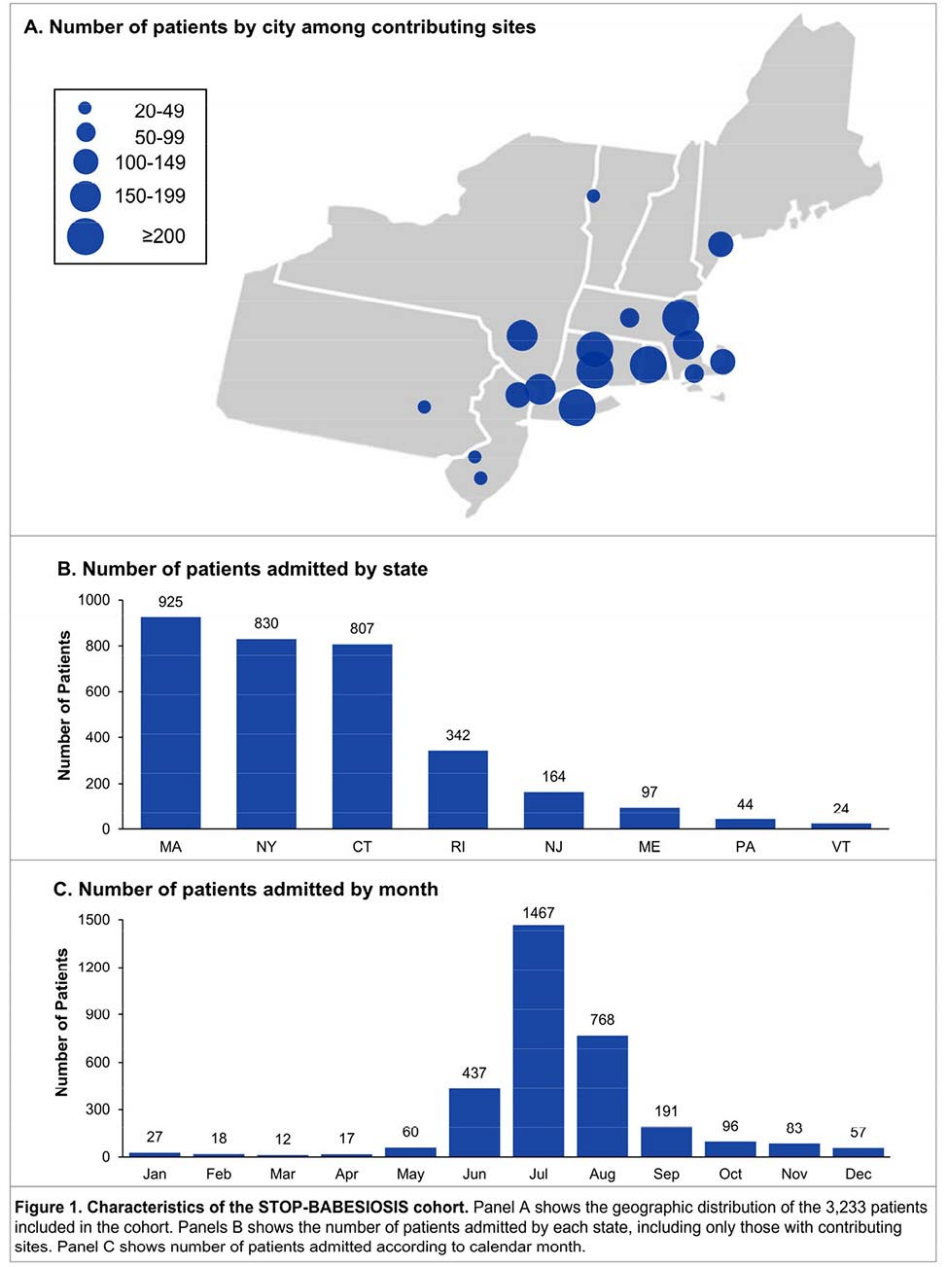

**Figure:**
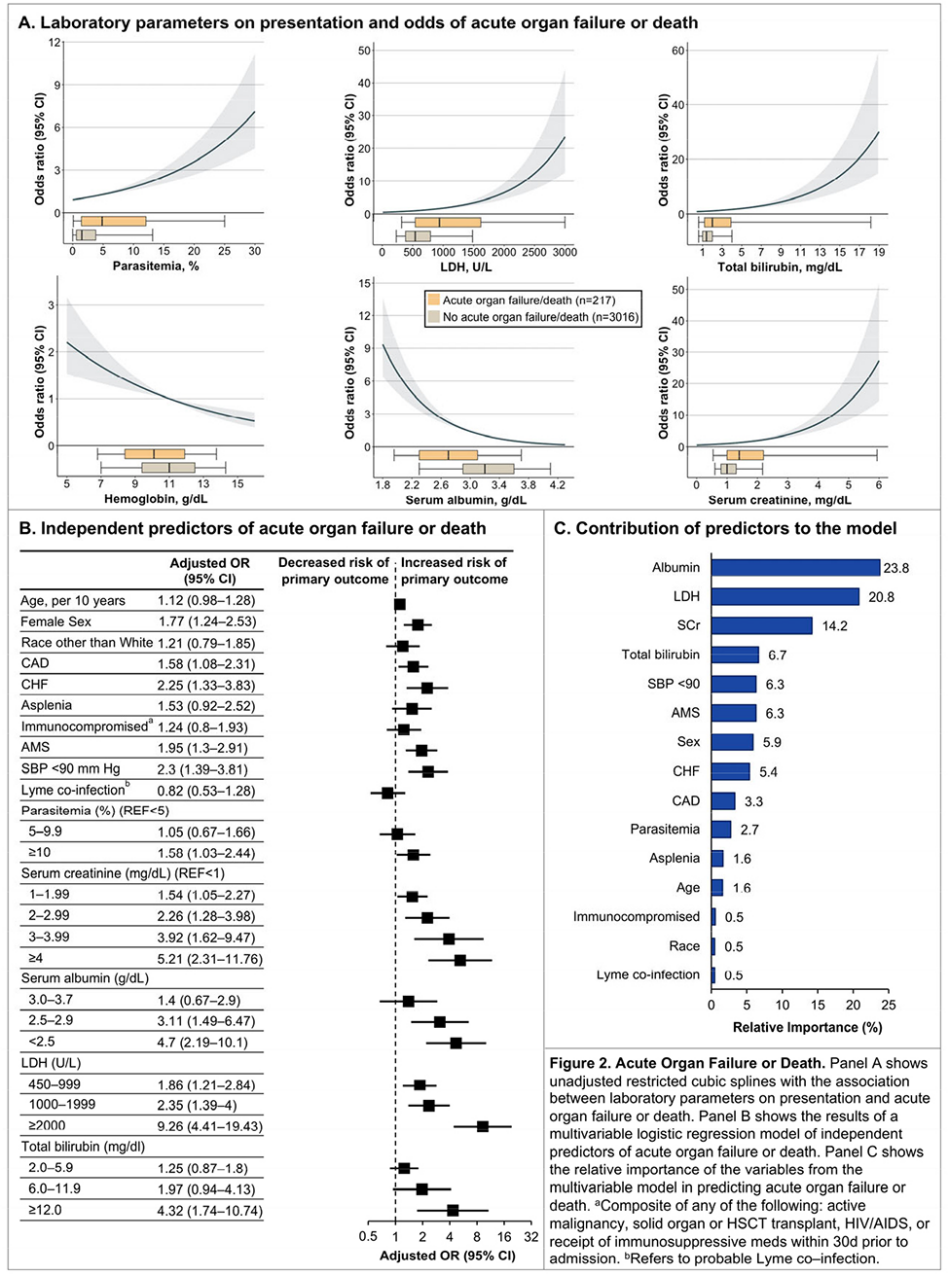

**Methods:**

We performed a multicenter cohort study of 3,233 consecutive adults hospitalized with babesiosis at 84 hospitals from 24 medical centers across 8 states in the northeastern US from 2010 to 2024. Data on demographics, comorbidities, vital signs, physiologic parameters, labs, treatments, and outcomes were collected by detailed chart review. Using multivariable logistic regression, we identified independent risk factors for the primary composite outcome of in-hospital death or acute organ failure. The latter was defined as shock requiring vasopressors, respiratory failure requiring invasive mechanical ventilation, or acute kidney injury requiring kidney replacement therapy. We also characterized the incidence and associated in-hospital mortality for a broader spectrum of acute organ injuries.

**Figure:**
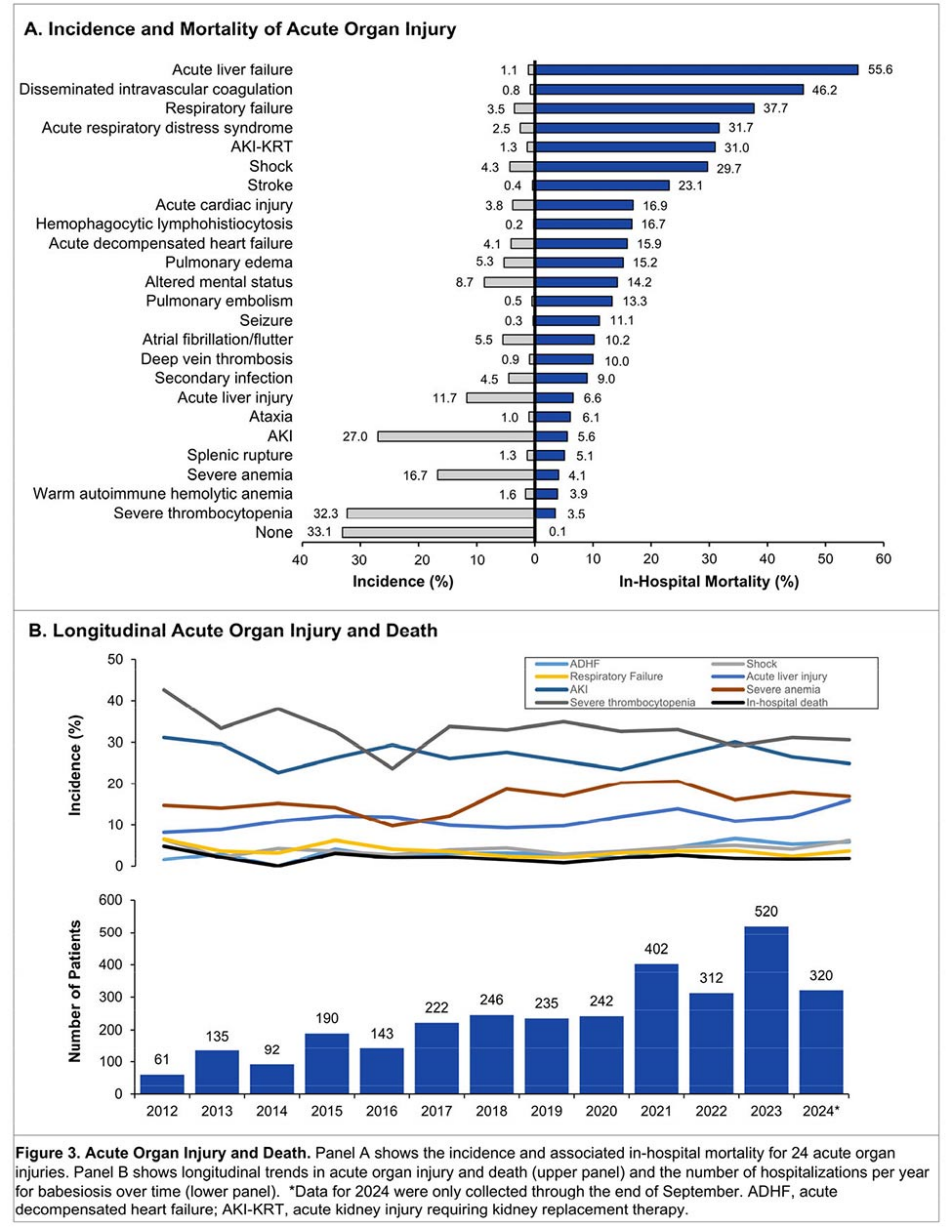

**Figure:**
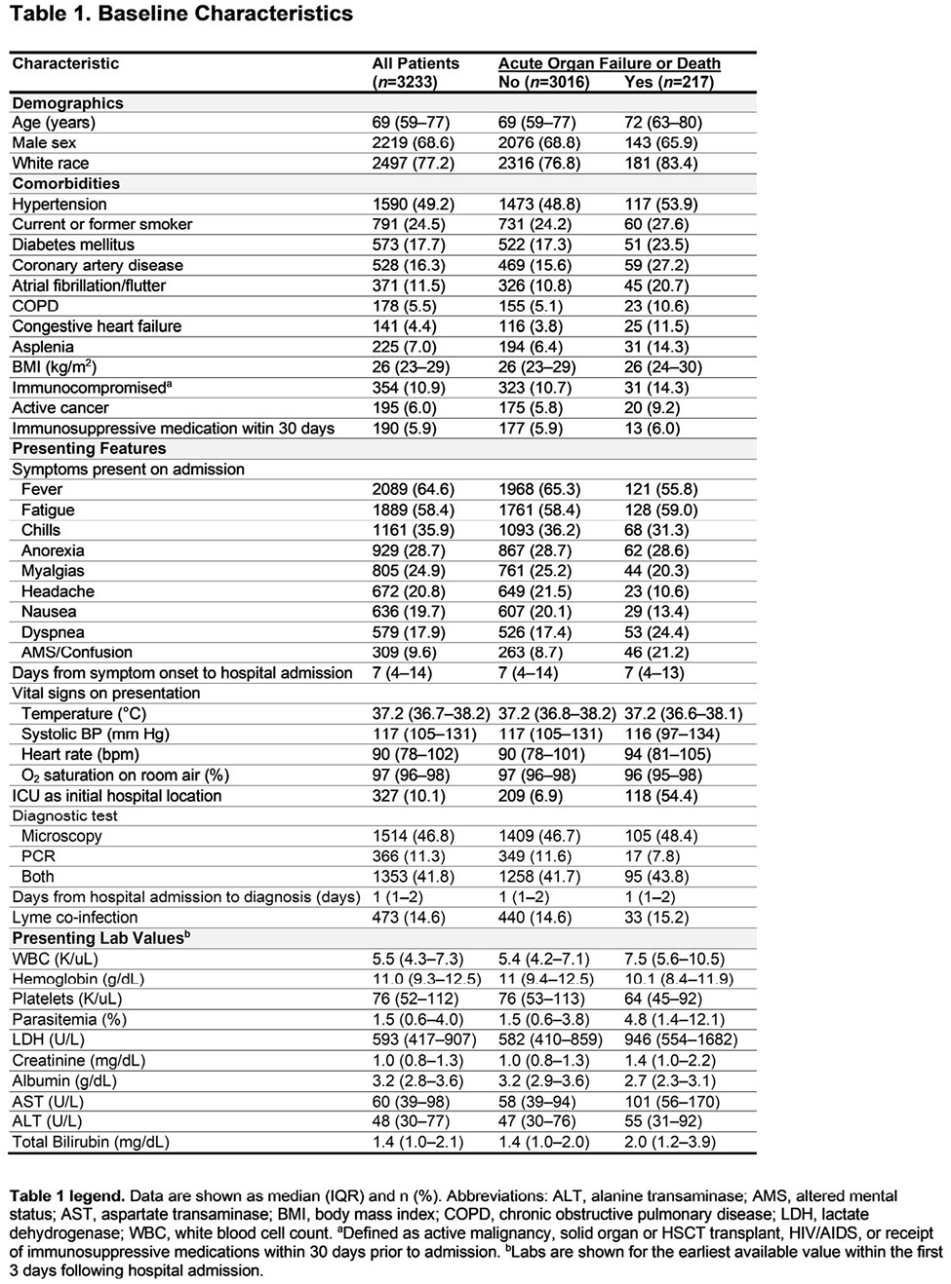

**Results:**

Characteristics of the 3,233 patients are shown in Figure 1 and Table 1. A total of 217 patients (6.7%) died or had acute organ failure. Higher parasitemia burden, LDH, total bilirubin, and serum creatinine, and lower hemoglobin and serum albumin on admission, were each associated with a higher odds of the primary outcome (Figure 2A). Ten independent risk factors for the primary outcome were identified (Figure 2B), with serum albumin, LDH, and creatinine having the highest relative importance (Figure 2C). A total of 2,163 patients (66.9%) developed ≥ 1 acute organ injury or failure during hospitalization (Figure 3A). Severe thrombocytopenia and acute kidney injury were the most common acute organ injuries, occurring in 32.3% and 27.0% of patients, respectively. Acute liver failure had the highest associated in-hospital mortality (55.6%), followed by disseminated intravascular coagulation (46.2%). The number of patients admitted per year with babesiosis increased over time, though the incidence of acute organ injury remained largely consistent (Figure 3B).

**Conclusion:**

This study identified demographic, clinical, and laboratory-based risk factors for in-hospital death or acute organ failure among hospitalized adults with babesiosis across a large number of sites and patients.

**Disclosures:**

Peter J. Krause, MD, 60 Degrees Pharmacueticals, Inc.: Grant/Research Support|Pfizer, Inc: Grant/Research Support David E. Leaf, MD, MMSc, Alexion Pharmaceuticals: Advisor/Consultant|Alexion Pharmaceuticals: Grant/Research Support|BTG International: Grant/Research Support|CardioRenal Systems, Inc: Advisor/Consultant|Metro International Biotech LLC: Grant/Research Support|Renibus Therapeutics, Inc: Grant/Research Support

